# An investigation of far and near transfer in a gamified visual learning paradigm

**DOI:** 10.1371/journal.pone.0227000

**Published:** 2019-12-26

**Authors:** Stefanie Duyck, Hans Op de Beeck

**Affiliations:** Brain and Cognition, Faculty of Psychology and Educational Sciences, University of Leuven (KU Leuven), Leuven, Belgium; Florida International University, UNITED STATES

## Abstract

After training, visual perceptual learning improvements are mostly constrained to the trained stimulus feature and retinal location. The aim of this study is to construct an integrated paradigm where the visual learning happens in a more natural context and in parallel for multiple stimulus types, and to test the generalization of learning-related improvements towards untrained features, locations, and more general cognitive domains. Half the subjects were trained with a gamified perceptual learning paradigm for ten hours, which consisted of an orientation discrimination task and a novel object categorization task embedded in a three-dimensional maze. A second group of subjects, an active control group, played ten hours of Candy Crush Saga. Before and after training, all subjects completed a ‘near transfer’ orientation discrimination and novel object categorization task, as well as a set of ‘far transfer’ general cognitive and attentional tasks. During the perceptual learning tasks, two different stimulus features and two retinal location pairs were assessed in each task. For the experimental group, one stimulus feature and retinal location pair was trained, whilst the other one remained untrained. Both features and location pairs were untrained in the control group. Far transfer did occur in some domains across all subjects irrespective of the training regimen (i.e. executive functioning, mental rotation performance, and multitask performance and speed). Near transfer was present in both groups, however only more pronounced for one particular task in the experimental group, namely novel object categorization. To conclude, all but one near transfer task did not generalize more than the control group.

## Introduction

Usain Bolt is a world champion expert at running 100 and 200 meters. Recently he quit athletics and strengthened a first class soccer team. Could Usain Bolt be the number one in cycling, chess and musical instruments too? Probably not: no one is born an expert, according to Malcolm Gladwell [1] and based upon a wide range of research studies [2] it takes about 10 000 hours or ten years of deliberate practice of one topic to reach the expert level. If expertise were domain specific, how could Usain Bolt play at a national soccer level lacking the specific soccer training all other players in the team received? This is the question which we aimed to address, not by using Usain Bolt, but the domain of visual perception.

For over forty years, visual perceptual learning (VPL) expertise has been a major interest of a broad range of researchers. Visual perceptual learning is characterized by training or experience with a specific visual stimulus in a specific task (e.g., Gabor patches in an orientation discrimination task). This training results in improvements in the perception/task performance for that specific stimulus [3–6]. The improvements were mainly found for the trained stimulus feature, retinal location, task, and sometimes even the trained eye, and often did not generalize to other features/locations/tasks/eyes [7]. This specificity was likely due to an overly rigid training regimen [8], which led to inflexible improvements.

Over the years various visual learning paradigms were documented to result in more general improvements, which we review here briefly in order of increasing distance from the classic perceptual learning paradigms. First, studies reported more general improvements after altering particular aspects of the classic paradigms [3,9,10]. Flexible performance gains were reported by Xiao et al. [11] as a result of a non-rigid double training paradigm. They conducted a classic contrast sensitivity experiment along with the training of a second, but irrelevant orientation discrimination training at a second location. Their results showed improved contrast sensitivity at both the trained and the second, irrelevant location. This type of within-domain (i.e. VPL) generalization is further referenced as ‘*near transfer*’ improvements. Since this study, several other studies reported similar effects [12–15]; but see [16–18] for some failures. In contrast to all previous perceptual learning studies, these researchers did not focus on one single stimulus or retinal location, which in turn apparently opened the door to generalization.

Second, beyond these flexible near transfer gains in the trained domain (e.g., VPL), studies observed broader real-life improvements, hereafter referenced as ‘far transfer’, after vision training, in reading abilities [19] and even baseball performance [20]. Furthermore, visually impaired individuals were shown to benefit from vision training, such as documented for amblyopia [21], presbyopia [22], and stroke [23].

Third, in 2003, Green and Bavelier [24] showed that where typical perceptual learning approaches failed to generalize learning, action video game play succeeded. More specifically, video game players outperformed novices on spatial and temporal attentional capacities. Moreover, to rule out a population bias, a group of novices was trained with an action video game and a control group played Tetris. Again, the action video game trainees showed enhancements in their spatial and temporal visual attention. According to a recent meta-analysis [25] on the impact of action video games on perceptual, cognitive, and attentional performances, generalization is mostly guaranteed in the domains of spatial cognition (e.g., mental rotation) and top-down attention (e.g., multiple object tracking).

Further articles proposed building blocks of video games that enhanced learning and created opportunities for generalization beyond the scope of the game. Elements such as *multiple stimulus types* [26], *various sensory systems* [27], *degree and intensity of exposure* [28], *task difficulty during training* [29] *and testing* [4], *presence of attention and reinforcement* [30] optimized the learning environment to obtain generalization. In addition to generalization, these factors also improved the degree [31,32] and speed [32] of learning.

Deveau et al. [26] argued that research on perceptual learning needs to integrate these learning optimization strategies instead of focusing on the separate learning strategies. They were the first to integrate all these building blocks into a perceptual learning game [26]. They developed a contrast sensitivity learning game, integrating a diverse stimulus set (i.e. multiple spatial frequencies, orientations and retinal locations), multisensory actions (i.e. low and high pitch sounds were used to cue the target’s location), exposure based learning (i.e. flickering stimuli at 20 Hz), and motivational assets (i.e. providing points and bonuses). They showed that professional baseball players, who played the game for 32 sessions, improved in contrast sensitivity, vision and even better baseball game performance (i.e. less strike-outs and more runs). Consequently, this perceptual learning game’s performance was apparently transferable to the real world.

These results were welcomed by many researchers: enhancing cognitive capacities just by playing videogames. Unfortunately, concerns were raised about the reproducibility of these findings. A recent meta-analysis by Sala and Gobet [33] made critical remarks about the inconsistent findings in experimental training studies, reviews and meta-analyses. More specifically, they argued [34] that a misconception of the results, placebo responses, and a publication bias cause these inconsistent results. Moreover, they claimed that when proper statistical modeling was applied, there might be no effect in the literature at large. As for the aforementioned meta-analysis [25], they reported generalization effects which would, as suggested by the authors, likely disappear after a publication bias correction.

In the present study we aimed to provide further evidence about the specificity of visual learning effects by developing and testing a paradigm that integrates features of more classic perceptual learning paradigms with a gaming environment that consists of the aforementioned building blocks. We therefore gamified a low- and a high-level visual feature perceptual learning experiment: orientation discrimination [35] and novel object categorization [36,37]. This innovative learning paradigm was used to test near transfer across perceptual manipulations, such as to other stimuli (e.g., novel orientations or objects) and other retinal locations (e.g., various locations at 5 degrees of visual angle from a central fixation cross), as well as far transfer to a wider set of cognitive functions (e.g., attention, multiple object tracking, & delayed recognition).

## Materials and methods

### Participants

Thirty-two healthy, non-gamer subjects were recruited through social media and a poster at the faculty of psychology at KU Leuven. All subjects received a monetary reward of eight euros per hour and gave written informed consent to participate in this study, which was approved by the social and societal ethics committee of the KU Leuven. Non-gamers were defined as having little to no PC or console gaming experience, however the inevitable occasional smartphone game (e.g., Pokémon Go) was allowed. Sixteen subjects were randomly assigned to the experimental group (12 females, *M*(age) = 22.13 years, *SD* = 2.19) and sixteen to the control group (13 females, *M*(age) = 22.50 years, *SD* = 2.22). The subjects had normal or corrected-to-normal vision and reported no neurological or medical problems.

### Apparatus

Novel objects were created using Matlab 2017b (MathWorks, Natick, MA) with Psychtoolbox. The gratings were generated in Python using the Psychopy version 1.9 toolbox (Peirce, 2009). For the testing procedure, a Dell PC running Matlab 2016b and Psychtoolbox was used to conduct the experiment. Subjects were placed in front of a DELL monitor with a 1600x900 resolution at 70 Hz. The training paradigm was made with Unity^®^ (version 2017.2.03f). For the training procedure, subjects used their personal computer running on Windows. For the course of the experiment, subjects were asked to uphold an upright position and to limit forward and backward movements from the screen during testing and training.

### Training phase

#### Experimental game

**Fig 1:**
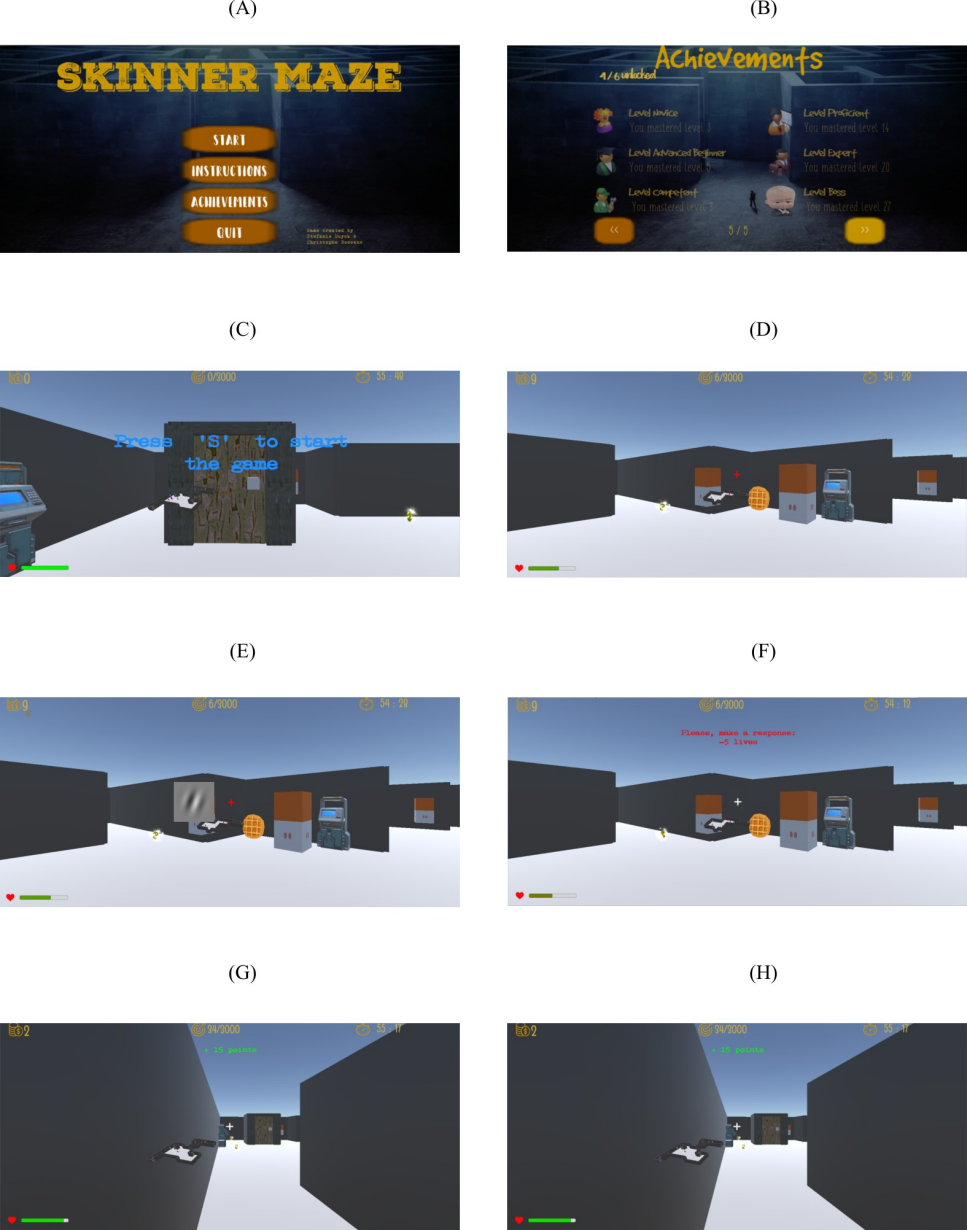
Screenshots from the game. **(A)** The main screen where subjects can start the game, read the instructions, look at their achievements or exit the game. **(B)** There are six different achievement pages regarding: collected cues, and coins, traveled maze distance, killed rats, and reached levels. Within each achievement, there are six different degrees: novice, advanced beginner, competent, proficient, expert, and boss. **(C)** At the start of each level, the subject is dropped somewhere in the maze. **(D)** Screenshot during the game. The pink-grey boxes represent the cues. After collection each cue was predictive (i.e. 70% probability) of the identity of the next stimuli. For example, when participating in the orientation discrimination task, two dots were likely followed by a clockwise rotation. Right before stimulus onset the white fixation turns red and subjects could not navigate. **(E)** Screenshot during stimulus presentation during which the subject could not navigate and had time to make a response. **(F)** The subject is punished for not responding or responding incorrectly and **(G)** rewarded for answering correctly. **(H)** During the game, the player received some knowledge on this position in the maze by means of the little room underneath the fixation cross, which only shows the nearby room(s) and collectibles.

The task paradigm (Fig 1) was designed to incorporate different learning mechanisms, such as rewarding environment, increasing task difficulty, and multiple stimulus and task sets. The paradigm consisted of a three-dimensional maze game through which the player had to navigate by means of a computer mouse (i.e. determine direction) and keyboard arrows (i.e. move forward, backward, left and right). The maze layout is randomly generated and the same during the entire experiment. In the instructions, players are told that they are captured by an evil professor, but that by helping to solve small challenges, the professor will set them free.

These small challenges were part of two perceptual learning tasks. The training paradigm included a novel object categorization and an orientation discrimination task, designed to probe representations at different levels of the visual processing hierarchy. In the even levels, subjects were challenged by a novel object categorization task (i.e. category 1 or category 2 smoothie/cubie). During odd levels, players had to participate in an orientation discrimination task (i.e. clockwise or counterclockwise to the 0° or 90° orientation reference).

**Fig 2:**
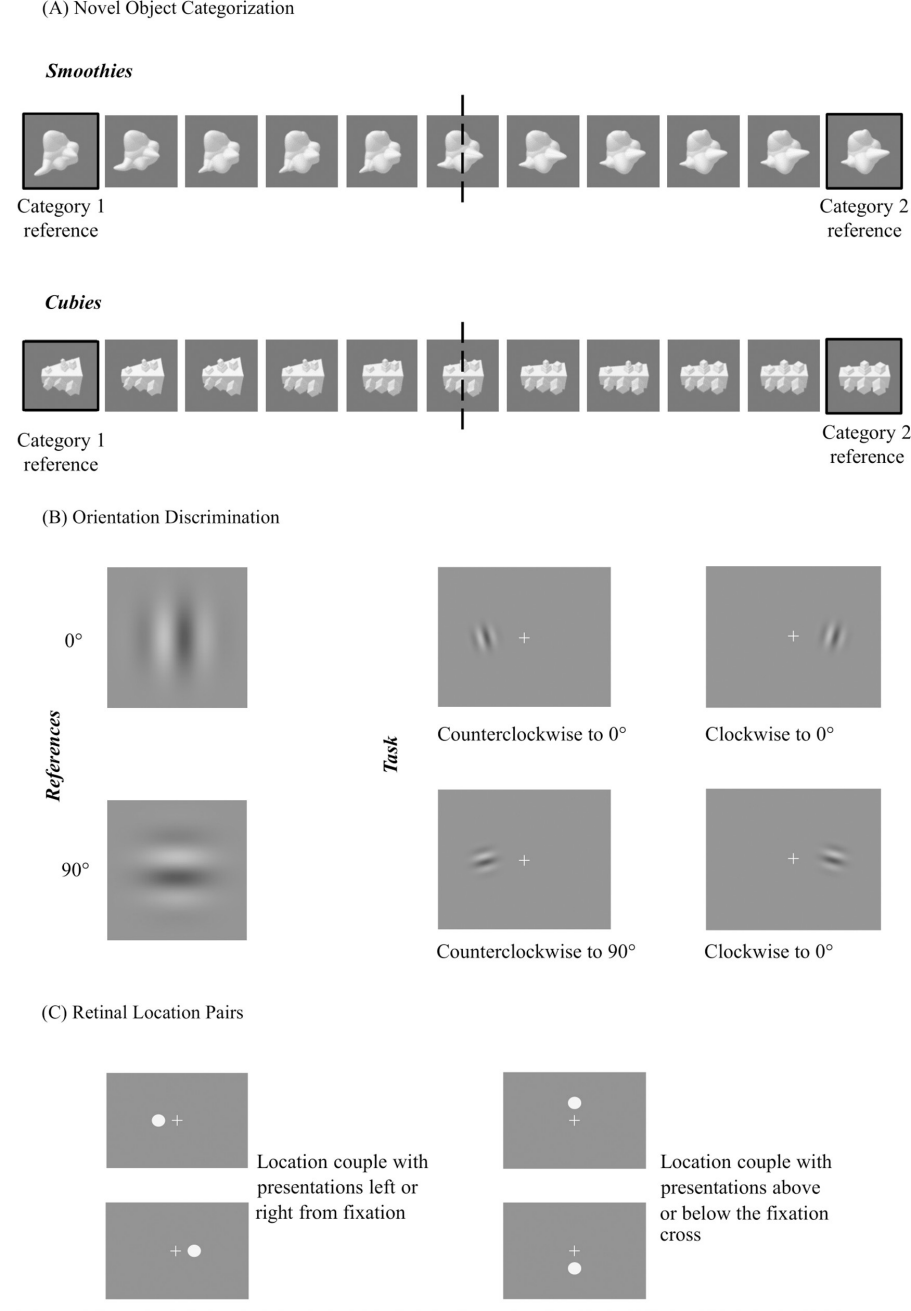
An overview of the perceptual stimuli, tasks, and retinal locations. **(A)** Demonstration of both object types (smoothies and cubies) and their subdivision between a category one and two object type. **(B)** Illustration of the 0 and 90 degrees Gabor references on the left and the task on the right. **(C)** Graphical display on the left of the left-right location pair, and above-below location pair on the right.

*Novel object categorization task*—In this task, subjects were instructed to maintain fixation on the central fixation cross while a novel object stimulus was presented for 150 ms (ISI sampled from a uniform distribution [5–7 seconds]) at five degrees eccentricity in one of two possible locations. The white fixation cross turned red for 500 ms right before the stimulus presentation to alert the subject. As illustrated in Fig 2C, two retinal location pairs (left-right and above-below) were used, such that across training overall, one pair of retinal locations was trained, but only one location per trial. We worked with a pair of locations symmetric with respect to the fixation point rather than one location to avoid that participants would be able to predict the presentation location. We used two object types [37] (Fig 2A), “smoothies” and “cubies”. For each object type, there are 1296 exemplars created by varying four dimensions (e.g., base shape and length protrusions) with six possible values on each dimension. We made two subcategories in each object category, with the reference stimuli of the two subcategories being the stimuli with either value zero or value five on all four dimensions. This division was explained before in Brants et al. [36]. By summing all dimension values of an object exemplar, which gives a sum of respectively 0 and 20 for the two reference stimuli, an exemplar belongs to the reference-0 subcategory when the sum is below 10 and to the reference-20 subcategory when the sum is above 10 (Fig 2A). During the task, subjects had to decide upon the subcategory of the shown object during a 2AFC staircase procedure (three down, one up), with step size equal to one sum value closer or further from 10. Each run/staircase had three dummy reversals and six experimental reversals, only the latter were included during the analyses. The average of each run’s six experimental reversals was used as the categorization threshold.

*Orientation discrimination task–*Similar to the object categorization task, subjects maintained fixation, but here one Gabor was presented each run. Subjects had to decide whether the shown stimulus was rotated clockwise or counterclockwise to the reference orientation in that run (0 or 90 degrees), which was shown during the instructions. The Gabor stimuli (Fig 2B), consisted of sinusoidal gratings with a Gaussian mask, extending over 3° of visual angle, contrast sampled from a uniform distribution [0.30 to 0.67], and spatial frequency sampled from a normal distribution (mu = 1.025; sigma = 0.01). Discrimination thresholds were measured using a 2AFC staircase procedure (three down, one up) with step size 0.05 log units to acquire a 79% convergence rate. Each staircase consisted of three dummy reversals, not included in the threshold analysis, and six experimental reversals of which the mean was calculated to obtain an orientation threshold.

Eight experimental conditions were counterbalanced between subjects: 2 Orientation references (0° or 90°) x 2 Retinal location pairs (Fig 2C) (left/right or above/below fixation cross) x 2 Object categories (smoothies or cubies). For each subject, one location pair was used in the training of the Gabor stimuli and the other pair for the objects. Throughout the game, subjects were only trained on one condition, for example 0° orientation reference as the trained orientation at the left and right from a central fixation cross together with the smoothies as the trained object category presented above and below the fixation cross.

The player started every level at a new maze location, from where they had to navigate through the maze and collect different items, such as coins and boxes. The collected coins could be used to buy extra player health or poison pills (to kill attacking rodents) at the corresponding vending machine, which was then spawned somewhere in the maze and signaled by smoke. The boxes were cues left by the professor to help the player by allowing them to predict the identity of the upcoming stimulus. Expectation tailors perception [38], such that top-down knowledge about the visual stimulus allows the subject to focus on the valuable information in the data jungle [39]. Moreover, this cue probability manipulation was shown to be of importance for obtaining transfer [40]. The cues were predictive of the upcoming stimulus feature’s orientation or category with a 70% probability.

At each difficulty level, a target amount of points was displayed, and a next level was entered when the subject reached the target. Players received points when they responded correct and lost player health when no response or an incorrect response was given. When subjects ran out of player health, they had to restart the current level. Consequently, finishing or failing a level could result in unfinished staircases. All data points were used to analyze the effect of predictive coding, but only the finished staircases were used to determine the orientation and categorization thresholds over time.

The game consisted of different levels with increasing task difficulty, such that the subjects needed to reach a higher amount of points, and coins and player health/poison pills vending machines were less easy to find. In addition, further features came into play, such as the presence of an attacking rodent against which players needed to be protected by buying poison pills. Subjects played the game on their personal computer at home for ten hours over the course of 14 days (one hour/day).

#### Control game–Candy Crush Saga

As a control game we used a popular puzzle game with no known nor expected benefits to everyday cognitive or attentional abilities: Candy Crush Saga [41,42]. The game was freely available on the internet, and subjects needed a working internet connection and a computer. Subjects had to register on the game’s website, in order to resume each day of training where they left off. During an episode of the Candy Crush Saga, the player needed to switch and combine different candies in a two-dimensional environment to collect points to get to the next level. There were multiple visual and auditory rewards for making delicious combinations. The different levels were characterized by increasing difficulty, novel candies and extras.

Note that in previous research, the puzzle game Tetris was often used to serve as a control training game [43,44]. Playing Tetris involved fast visual-motor control and resulted in improved spatial performances [45], however, to a lesser extent than action videogames did. Tetris players memorized the game’s object shapes and their spatial configurations, which are pretty similar capacities to a mental rotation task. As a result of this resemblance and the assessment of a mental rotation task in the test phase, we decided to use Candy Crush Saga instead.

### Test phase

**Fig 3:**
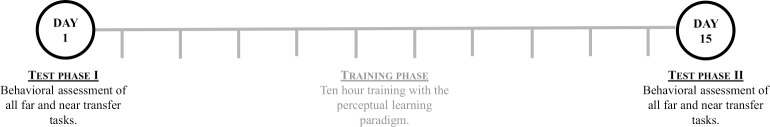
A visual timeline infographic of the study.

All tests were conducted before and after training in each participant, and were 14 days apart in time (Fig 3).

#### Near transfer

*Novel object categorization task–*Similar to training, subjects were instructed to maintain fixation on the central fixation cross while a novel object stimulus was presented for 150 ms (ISI 300 ms) at five degrees eccentricity in one of four possible locations. As illustrated in Fig 2C, two retinal location pairs (left-right and above-below) were used, such that during each run, one pair of retinal locations was assessed, but only one location per trial. There were four runs in total, one for each location pair (i.e. left-right and above-below fixation cross) (Fig 2C) and novel object reference (i.e. smoothie and cubie), counterbalanced across runs. The average of each run’s six experimental reversals was used as the categorization threshold for that stimulus and retinal location pair.

*Orientation discrimination task*–The task design was similar to the one used during training, such that subjects had to decide whether the shown stimulus was rotated clockwise or counterclockwise to the reference orientation in that run (0 or 90 degrees), which was shown during the instructions. There were four runs, such that each run consisted of one orientation reference and location pair, which was counterbalanced over runs. Each staircase’s six experimental reversals were used to obtain an orientation threshold.

#### Far transfer

We selected tasks of cognitive abilities that were implicated in previous studies of visual and cognitive training.

*Attentional Network Task–*This task [46] was administered to evaluate three domains of attention: alerting, orienting and executive functioning. Subjects were presented a set of arrows, which could either be above or below the central fixation cross. Beforehand, a cue could be presented for 100 ms to either alert (i.e. an asterisk presented on top of the fixation cross) or orient (i.e. an asterisk appeared below or above the fixation cross) the subject with regard to the upcoming target stimulus. The target stimulus, on screen for maximum 1700 ms, consisted of an arrow which was surrounded by either congruent or incongruent arrows or neutral dashes. There were eight blocks of 48 trials during which subjects needed to decide upon the direction (i.e. left or right) of the target arrow.

*Multiple Object Tracking Task*–The multiple object tracking task was designed to experimentally evaluate the subjects’ visual system’s performance to track multiple objects. The classic experiment showed a collection of dots on a computer screen, some of which would turn yellow. When returned to their standard color, all the dots started moving. When they stopped moving, subjects were asked to indicate where the previously colored dots were situated now. We applied the modified version by Green and Bavelier [44], which opted to limit the influence of working memory to obtain a more clear measurement of selective attention to objects. Instead of asking to indicate all the previously colored dots (1–7 dots), they presented the subjects with a display with only one highlighted dot and asked to decide in a yes or no fashion whether this dot was colored before the moving started. Subjects were presented static scattered dots (n = 16) of which one to seven dot(s) was colored red. After 2000 ms, all dots turned green and started moving (pace: five degrees per second) for 5000 ms after which they stopped and one dot colored white for 3000 ms. Subjects decided (yes or no) whether this white dot was among the red colored dots before the dots started to move.

*Mental Rotation Task–*Subjects were presented two three-dimensional shapes [47,48] which could differ in shape (i.e. same/mirrored) and angular disparity (i.e. 0, 50, 100, 150 degrees), and they needed to make a same/different shape response despite the angular disparity. A blank screen was shown for 250 ms followed by the presentation of two shapes for a maximum of 7500 ms. Two blocks of 48 trials (i.e. 24 same and 24 different trials) were conducted and each angular disparity occurred twelve times across these two blocks.

*Multi-tasking–*Two separate tasks [49], a visual and an audio categorization task, were administered on their own (each 60 trials) and intertwined (120 trials). During the visual categorization task one of three triangles, each with a different size (i.e. small/medium/large), was randomly presented for 400 ms. For the auditory task, one of three beep tones, each with a different frequency (i.e. 350/900/1650 Hz) was presented for 40 ms. Subjects had to categorize the stimulus by pressing the corresponding button.

*Test of Variables of Attention–*The test [50,51] assessed sustained attention by asking participants to make a go response when a little black box is presented for 100 ms in the upper half of a white box which is presented in the center of the screen, and a no-go response when it was presented in the lower half. There were two conditions: during the stimulating condition (last 306 trials), seven out of nine presentations required a go-response, whereas the boring condition (first 306 trials) consisted of twenty-two percent go responses.

*Cognitive Failures Questionnaire–*In addition to the foregoing experimental tests, we also obtained *self-reported measurements on failures in perception*, *memory and motor function*. The Cognitive Failures Questionnaire [52] measured self-reported failures in perception and memory and has not been administered before in the context of transfer in expertise training. The questionnaire contained 25 items (e.g., ‘Do you fail to hear people speaking to you when you are doing something else?’ and ‘Do you forget appointments?’) and subjects were asked to pick an answer (4 ‘very often’, 3 ‘quite often’, 2 ‘occasionally’, 1 ‘very rarely’ and 0 ‘never’) that best described the situation.

## Results

### Performance during training

#### Experimental game

In general, subjects reached on average game level eleven (*M* = 10.69, *SD* = 5.61), and finished about two levels each day of training (*M* = 1.95, *SD* = 0.51).

*Novel object categorization task–*During even game levels, subjects were trained on one object type (i.e. smoothie or cubie) at one location pair (i.e. above-below or left-right). Once they reached three dummy reversals and six experimental reversals, a staircase was finished. Each staircase provided a mean categorization threshold (i.e., number of transformations away from the category boundary (max 10)) by averaging these six experimental reversals values. Due to the gaming environment, there were cases where the subject did not finish a staircase, because of level completion or level failure. As a result, subjects were trained on average for 2707 trials, but the complete staircases only contained 1617 trials.

**Fig 4:**
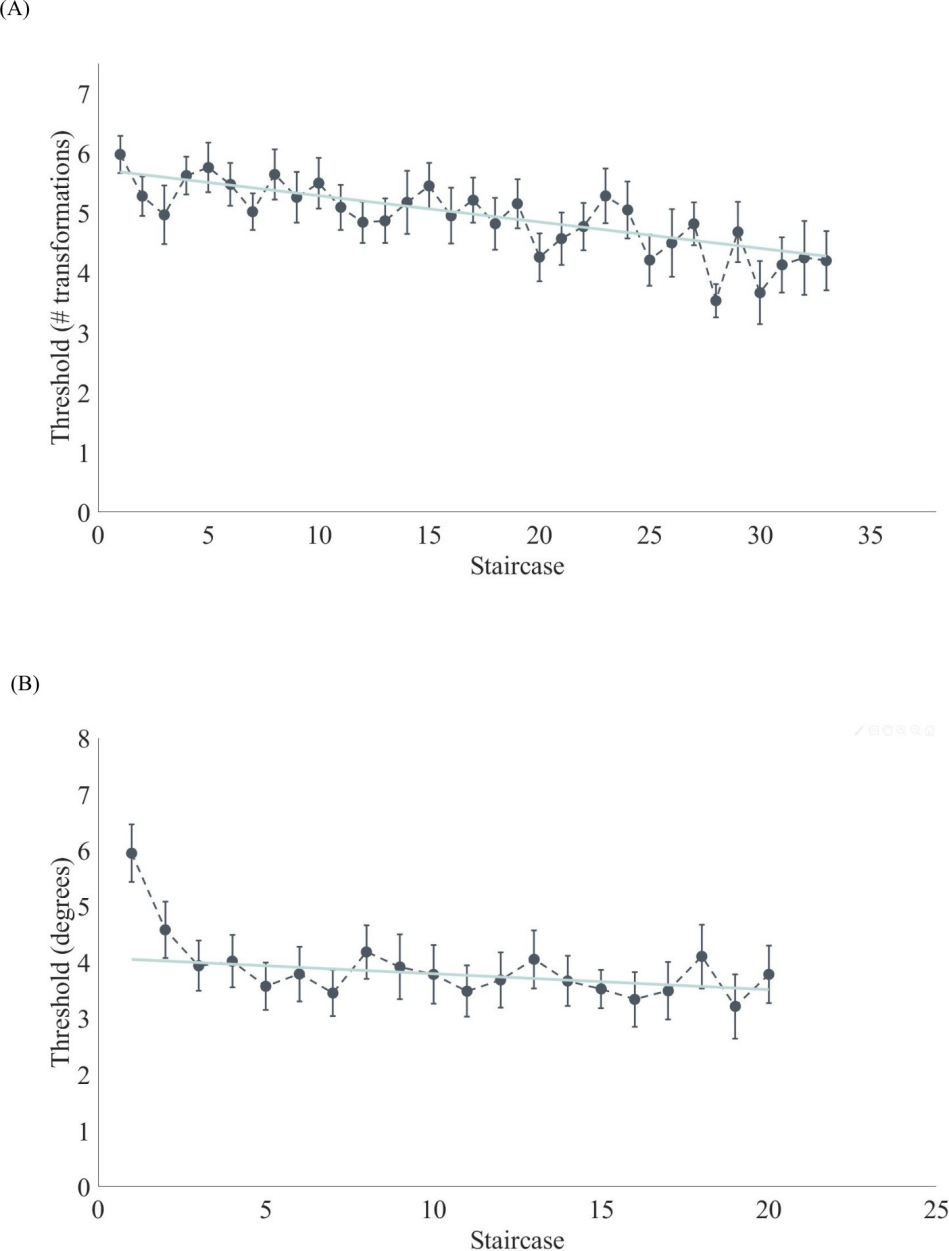
**A summary of the perceptual learning thresholds during the game in the novel object categorization task (A) and orientation discrimination task (B).** The error bars represent the standard error across all subjects for the average number of finished staircase. The green line represents the learning slope across subjects over the average number of staircases.

Given that the total amount of staircases differed across subjects, we fitted a linear model across the mean number of staircases (n = 33) across all subjects to model the relationship between the mean categorization threshold and the number of the staircase. Outliers were excluded after Cook’s distance analysis. The results showed learning over time (slope = -0.04, intercept = 5.73, *F*(1, 28) = 58.20, and *p* < .001), such that the categorization threshold decreased. Over practice time (Fig 4A), novel objects could be categorized which were transformed further away from the reference category and thus closer to the boundary between the two categories. Subjects obtained a mean convergence percentage of 80.64% across all runs.

Furthermore, the presence of predictive cues on the categorization performance was evaluated. If the subject made use of the hints, we expected a better performance with valid cue-target association versus invalid cue-target. By means of a two-tailed two sample t-test between valid and invalid cue-target performance, we showed no advantage of valid over invalid cues (*M*(valid) = 80.09%, *M*(invalid) = 80.40%, *t*(30) = -0.14, *p* = 0.888).

*Orientation discrimination task–*Throughout the maze game, subjects were trained in an orientation discrimination task with one reference orientation (i.e. 0° or 90°) and in one location pair orthogonal to the object’s location pair (i.e. above-below or left-right). The experimental reversals in each finished staircase were used to calculate an orientation threshold for each staircase. On average, each subject completed 1721 trials, which consisted of both finished and unfinished staircases (e.g., out of lives or level completion). For the following analyses only finished staircases were used, which resulted in 1167 trials on average per subject.

Similar to the analysis of the novel object categorization task, a linear regression analysis was carried out between the orientation threshold and the number of a staircase (mean number of staircases was 20 per subject). Outliers were excluded by means of Cook’s distance measure. A significant decreasing linear relationship was found with slope = -0.03°, intercept = 4.08°, *F*(1, 17) = 5.05, and *p* = .038. The results clearly indicated that the orientation discrimination threshold improved with practice. Across all runs, subjects obtained a mean convergence percentage of 83.51%.

Findings did not reveal cue learning, such that a two-tailed two-sample t-test indicated a non-significant difference between the orientation discrimination performance when a valid (*M* = 88.71%) compared to an invalid (*M* = 88.74%) cue was presented (*t*(30) = 1.34, *p* = 0.191).

#### Control game

The control game did not include perceptual learning tasks, but in order to have some measurement of training performance, the last accomplished level of each day of training was analyzed. To evaluate whether subjects reached higher levels with every day of training, a linear regression model was composed with day of training as predictor and last fulfilled level as outcome variable. The linear regression model showed a positive learning effect (slope = 5.71, *F*(1, 8) = 16, *p* < .001). To conclude, all subjects reached higher levels as training progressed.

### Performance during testing

#### Near transfer

*Novel object categorization task*–Both groups performed the novel object categorization task before and after game training. All combinations of object types and locations were tested. Thus, for the experimental group, both the trained and untrained object types were evaluated at both location pairs (trained and untrained). We express the results for each object and location in terms of the categorization threshold (number of transformations away from the category boundary (max 10)), computed from the experimental reversals at the end of the adaptive staircases.

At baseline, a two sample t-test revealed that the categorization threshold of the experimental group was not significantly different from the control group in both the smoothies (*M*(experimental group) = 7.00, *M*(control group) = 7.30, *t*(10) = 2.12, *p* = .060) and the cubies (*M*(experimental group) = 6.72, *M*(control group) = 6.76, *t*(10) = 0.26, *p* = .799). In the experimental group, the baseline categorization thresholds of smoothies and cubies were not significantly different from one another, as revealed by a two-tailed two sample t-test (*t*(10) = 1.49, *p* = .167). However, in the control group, there was a small noticeable baseline difference between the smoothies and cubies threshold (*t*(10) = 5.34, *p* < .001). Counterbalancing all training conditions across subjects avoids that this difference can influence our results, as an equal number of subjects were trained with smoothies and cubies.

A two-way, mixed ANOVA with session (pre- and post-training) as within-subject factor and group (experimental and control) as a between-subject measure on categorization threshold revealed a main effect of session (*F*(1, 30) = 38.23, *p* < .001), a significant interaction between group and session (*F*(1, 30) = 8.11, *p* = 0.008), and no main effect of group (*F*(1, 30) = 1.78, *p* = .192). This finding illustrated the presence of learning in both groups, but pointed to a non-robust learning gap between the experimental and the control group due to the absence of a group main effect. The specifics of learning were further analyzed via paired t-tests within each group (all p-values are one-tailed).

**Fig 5:**
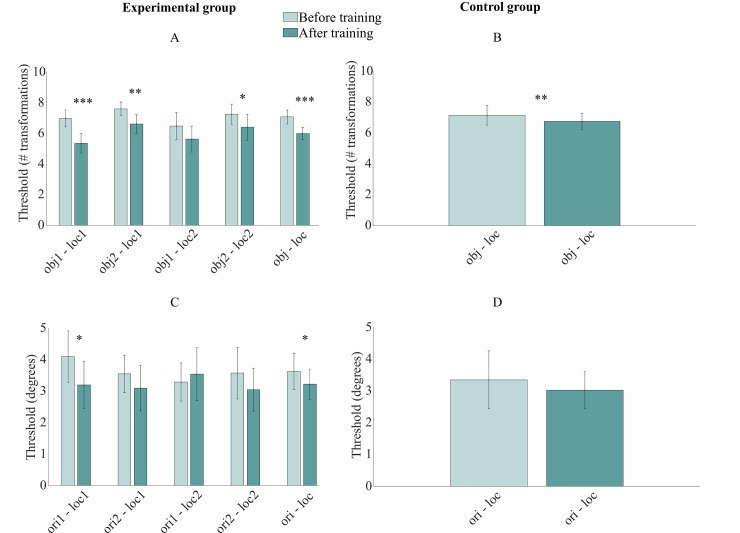
A summary of the near transfer results. The mean threshold in each condition and across all conditions before and after training in the novel object categorization task **(A-B)** and the orientation discrimination task **(C-D)**. obj1/ori1 = trained stimulus, obj2/ori2 = untrained stimulus, ori1 = trained location pair, ori2 = untrained location pair, obj/ori = trained and untrained conditions together, and loc = trained and untrained location pairs together. Error bars represent the standard error of the mean across participants. A significant difference between pre- and post-training is marked as * p < .05, ** p < .01, and *** p < .001.

In the experimental group (Fig 5A), there was an overall improvement from pre- to post-training (*M*(session 1) = 7.06, *M*(session 2) = 5.99, t(15) = -5.87, p < .001). Next, we investigated the specificity of learning by comparing the performance of each object+location condition pre- and post-training. As expected, there was a clearly improved categorization in the trained object at the trained locations (t(15) = -5.07, p < .001). Furthermore, a significant improvement was observed for two transfer conditions: the untrained object at the trained locations (t(15) = -3.56, p = .001), and the untrained object at the untrained locations (t(15) = -1.83, p = .043). There was no significant difference for the trained object at the untrained location (t(15) = -1.64, p = .061).

The control group (Fig 5B) too showed learning as a result of the control game, as revealed by the one-tailed paired t-test over all conditions (*M*(session 1) = 7.15, *M*(session 2) = 6.75, t(15) = -2.61, p = .010).

To conclude, both the experimental group and the active control group improved their categorization threshold after training, although not to the same degree. Subjects trained with the perceptual learning maze paradigm showed more, although non-robust, learning compared to the active Candy Crush group. Furthermore, the improvement in the experimental group transferred to some of the untrained conditions.

*Orientation discrimination task*–We assessed the subject’s performance to discriminate orientations in two base orientations, of which one was trained (i.e. 0° or 90°) and one orthogonal (i.e. 90° or 0°) to the trained orientation, and two location pairs of which one trained and one untrained (i.e. above-below or left-right). Performances were evaluated by averaging the six experimental reversals of each run, resulting in an orientation threshold in degrees for each base orientation at each location pair.

A two-tailed two sample t-test showed a small difficulty difference between the orientation references. This difference in difficulty between both orientation references was present in both the experimental and the control group, such that orientation discriminations around the 90° reference were a bit harder to make (Mean threshold experimental group (90°) = 3.53°, *M*(0°) = 3.04°, *t*(10) = 2.27, *p* = .046 and Mean control group (90°) = 3.24°, *M*(0°) = 2.68°, *t*(10) = 2.63, *p* = .025). However, this is not a problem given that all conditions are counterbalanced between subjects. Moreover, there are no differences at baseline between both groups within each reference (90°: *t*(10) = 1.16, *p* = .275 and 0°: *t*(10) = 2.01, *p* = .062), confirming that the two groups were matched prior to learning.

In order to observe differences in the mean threshold before and after training between groups and sessions, we conducted a two-way, mixed ANOVA with session as within-subject variable and group as between-subject measure on orientation discrimination threshold. The analysis showed a main effect of session (*F*(1, 30) = 6.12, *p* = .019) on orientation threshold, no significant main effect of group (*F*(1, 30) = 0.15, *p* = .701), nor a significant interaction between group and session (*F*(1, 30) = 0.91, *p* = .765).

Learning from pre -to post-training was further analyzed by means of one-tailed paired t-tests for all conditions individually in the experimental group and one test for all conditions combined in both groups. In the experimental group (Fig 5C), subjects improved on the trained orientation at the trained location (*t*(1, 14) = 1.99, *p* = .034) and over all conditions (*M*(session 1) = 3.78°, *M*(session 2) = 3.19°, *t*(15) = 1.87, *p* = .041). In the control group (Fig 5D), the test across all conditions revealed a non-significant difference from pre- to post-training (*M*(session 1) = 3.57°, *M*(session 2) = 3.11°, *t*(15) = 1.63, *p* = .062).

These results illustrated a small general learning effect in the experimental group. The absence of a training effect specific to the trained group was likely the result of insufficient statistical power. Due to the game environment we were not able to control for the total amount of trials in each task, and as a result, there were more trials during the object categorization task. Additionally, the pre to post behavioral perceptual learning data of one subject was incomplete. To conclude, the orientation discrimination findings showed more learning specificity in contrast to the transferred object categorization results.

### Far transfer

*Attentional Network Task*–Before conducting any statistical tests, reaction time data from incorrect trials were excluded. Similar to Dye, Green & Bavelier (2009), we checked whether there were trials with unusual slow responses within each condition, but there were no trials with reaction times more than two standard deviations from the condition (4 (Cue type) x 2 (Flanker)) average. Additionally, median reaction times were entered in all statistical tests.

The attentional network analysis focused on four important outcome measures (in line with previous studies): median reaction time, alerting score, orienting score, and executive control score. First, a four-way mixed ANOVA was performed on the median reaction time data with session, flanker type (incongruent, congruent) and cue type (absent, center, double, spatial) as within-subjects factors, and group as between-subject factor. A main effect of session (*M*(session1) = 0.55 s, *SD* = 0.08, *M*(session2) = 0.51 s, *SD* = 0.06, *F*(1, 30) = 61.65, *p* < .001) was taken as a general effect of learning, however, the absence of an interaction between group and session (*F*(1, 30) = 0.52, *p* = .475) showed that the learning effect was not specific to the experimental group. Furthermore, a main effect of group suggested a discrepancy in median reaction times across all within-subject factors between groups (*M*(experimental group) = 0.50 s, *SD* = 0.08, *M*(control group) = 0.55 s, *SD* = 0.06, *F*(1, 30) = 7.56, *p* < .001).

Second, the alerting score reflected the capability of benefitting from a temporal cue (an asterisks at each possible target’s location) when making decisions about the target. These individual scores were defined by the no cue condition minus the double cue condition median reaction times. Third, an orienting score quantified the advantage of a validly cued spatial location over a location irrelevant (central fixation) cue. Last, executive functioning score was the result of the degree of distraction/help caused by incongruent/congruent flankers on target assessment. All scores had a value different from zero, which validated the paradigm. For each of the just described ANT scores, a two-way mixed ANOVA was computed with session as within-subject factor and group as between-subject factor. A main effect of session was shown for the executive performance (*M*(session1) = 0.09 s, *SD* = 0.03, *M*(session2) = 0.08 s, *SD* = 0.02, *F*(1, 30) = 11.38, *p* = 0.002), and a non-significant result was found in the ability to orient (*F*(1, 30) = 4.09, *p* = .052). There was no main effect of session for alerting (*F*(1, 30) = 0.09, *p* = .766). No significant main effect of group (alerting *F*(1, 30) = 1.32, *p* = .260; orienting *F*(1, 30) = 2.16, *p* = .152; executive functioning *F*(1, 30) = 2.05, *p* = .163) or Session x Group interaction effects were found (alerting *F*(1, 30) = 1.13, *p* = .295; orienting *F*(1, 30) = 2.87, *p* = .100 ; executive functioning *F*(1, 30) = 0.63, *p* = .432).

To conclude, subjects improved their median reaction times and executive functioning independent of the type of training (i.e. perceptual learning maze or Candy Crush Saga) they received.

*Multiple Object Tracking Task*–The effect of training on the performance to track multiple objects was evaluated by means of a three-way mixed ANOVA with session and number of colored dots as within-subject variables and group as between-subject factor. Results showed no main effect of session (*F*(1, 30) = 0.10, *p* = .752), group (*F*(1, 30), *p* = .35) nor an interaction effect between session and group (*F*(1, 30) = 0.07, *p* = .792). The intensive training paradigms did not elicit changes in the ability to track multiple objects.

*Mental Rotation Task*–A three-way mixed ANOVA with session and rotation degree (0–50–100–150) as within-subject variables and group as between-subject factor indicated a main effect of training (*M*(session1) = 79.38%, *SD* = 0.19, *M*(session2) = 85.99%, *SD* = 0.19, *F*(1, 26) = 9.39, *p* = .005), but no Session x Group interaction (*F*(1, 26) = 1.12, *p* = .299) on the mental rotation performance. Additionally, subjects did not get faster after training: no main effect of session (*F*(1, 26) = 22.51, *p* = .606) or Group x Session interaction (*F*(1, 26) = 0.00, *p* = .964) on the mental rotation reaction time.

*Multi-tasking*–The ability to perform two different tasks in rapid alternating sequence improved after training, but did not differ between training regimes. This was confirmed by a three-way mixed ANOVA with session and task type as within-subject variables and group as between-subject variable on the task performance. There was a main effect of session (*M*(session1) = 32.44%, *SD* = 35.80, *M*(session2) = 35.64%, *SD* = 37.83, *F*(1, 30) = 5.25, *p* = .029), but no interaction between session and group (*F*(1, 30) = 0.13, *p* = .722). Subjects did become faster after training (*M*(session1) = 0.84 s, *SD* = 0.12, *M*(session2) = 0.85 s, *SD* = 0.11, *F*(1, 25) = 5.08, *p* = .033), but this was independent of the received training (Session x Group: *F*(1, 25) = 0.98, *p* = .332), as shown in the three-way mixed ANOVA with session and task type as within-subject factors and group as between-subject factor on the mean reaction times of correct responses.

*Test of Variables of Attention*–In line with previous studies, the analysis of the TOVA with relation to video games was fourfold: response speed, impulsivity, sustained attention, and sensitivity index. First, response speed was defined as the mean reaction time of correct trials. Second, impulsivity or the error of commission was measured as the amount of go responses where no response was allowed. Third, sustained attention or the error of omission, which was the error of not making a go response when one was required. Fourth, the sensitivity index or d’ is a well-known measure which subtracts the z-score of the false alarm rate from the hit rate.

All four dependent measures were entered into a four-way, mixed ANOVA with session, condition (stimulating/boring) and quarter (first, second, third or fourth quarter of trials) as within-subject variables and group as between-subject factor. Across these four measures, no main effect of session (mean reaction time *F*(1, 29) = 0.57, *p* = .455; impulsivity *F*(1, 30) = 3.54, *p* = .070; sustained attention *F*(1, 30) = 1.50, *p* = .231); d’ *F*(1, 30) = 0.63, *p* = .433). Furthermore, no Session x Group interaction was visible (mean reaction time *F*(1, 29) = 1.66), *p* = .207; impulsivity *F*(1, 29) = 0.91, *p* = .347; sustained attention *F*(1, 30) = 0.03, *p* = .857); d’ *F*(1, 30) = 1.71, *p* = .201). To conclude, subjects’ game play did not alter impulsivity or attention capacities.

*Cognitive Failures Questionnaire*–In addition to all previous tasks, the cognitive failures questionnaire was administered to obtain a self-reported measure on the subject’s cognitive flaws. There are twenty-five questionnaire items used for a total cognitive functioning score, with seventeen of them over four subscales: distractibility, forgetfulness in social situations, forgetfulness in names and words, and forgetfulness in orientation. Each item is scored on a five-point scale, ranging from 0 (very often) to 4 (never) and the reversed scores are summed to obtain the total cognitive functioning score and its sub scores. A two-way mixed ANOVA with session as within-subject variable and group as between-subject variable showed no effect of training on the total score (session: *F*(1, 30) = 1.49, *p* = .232; Session x Group: *F*(1, 30) = 0.20, *p* = .661). Furthermore, there was no evidence of training effects (i.e. main effect of session) on the four sub scores as dependent variables: distractibility (*F*(1, 30) = 0.34, *p* = .563), forgetfulness in social situations (*F*(1, 30) = 0.26, *p* = .613), forgetfulness in names and words (*F*(1, 30) = 1.18, *p* = .285), and forgetfulness in orientation (F(1, 30) = 1.47, *p* = .234). Additionally, no interaction between session and group was found: distractibility (*F*(1, 30) = 1.37, *p* = .252), forgetfulness in social situations (*F*(1, 30) = 0, *p* = 0.999), forgetfulness in names and words (*F*(1, 30) = 0.13, *p* = .720), and forgetfulness in orientation (*F*(1, 30) = 0.27, *p* = .607). To conclude, subjective cognitive functioning did not show any alterations as a result of the training.

## Discussion

In the present study, we created and tested an innovative perceptual learning paradigm which contained learning optimization features similar to the building blocks of videogames. This paradigm was a prime candidate to investigate the specificity of learning in two domains: perceptual learning (i.e. near transfer) and cognitive functions (i.e. far transfer). The study presented two main findings. First, both the experimental group and the active control group showed learning in both domains as a result of training. Second, in one of the two perceptual learning tasks, the degree of improvement depended upon the type of training, such that the perceptual learning paradigm elicited more learning than the active control game. Third, as far as learning was observed in far transfer, it was not different between the experimental group and the active control group.

### Near transfer

Half the subjects were trained with the perceptual learning paradigm, which contained a novel object categorization task and an orientation discrimination. For the first task, each subject was trained on one object type (i.e. smoothie or cubie) at a particular retinal location pair (i.e. left-right or above-below fixation at 5° from central fixation cross). Findings showed that both the active control group and the experimental group enhanced their capacity to categorize novel objects in general (trained and untrained Object type x trained and untrained Retinal location pair). These results indicated that both games were able to elicit learning, or that more general test-retest effects play a role. Furthermore, both groups did not improve to the same degree: we found better performance across all conditions for subjects trained with the perceptual paradigm. During the second task, one orientation reference was trained at the retinal location pair orthogonal to the location pair used for the object categorization task. The performance to discriminate improved after training solely in the experimental group, although less pronounced than was the case in object categorization.

We also investigated the specificity of the training effects in each perceptual learning task. Inherently connected to perceptual learning, are improvements in the trained features at the trained locations, which were clearly present in this study. In the second test phase, results showed improvements in the trained object type and orientation reference at their trained retinal locations. However, the novel object categorization findings also revealed gains in the performance of untrained features in both trained and untrained locations, but no progress in trained features in untrained locations. In the orientation discrimination task we lack the power to investigate specificity.

The game environment might be a factor that influences specificity. According to Herzog et al.[53], multiple different manipulations have it in their power to challenge learning specificity. The most popular ones are the double training [11] and the training-plus-exposure [14] paradigm. However, different from these paradigms, our paradigm did not in the same way make use of a relevant secondary task, nor similar stimulus features. Following Wang et al. [54] transfer only occurred during the presence of a relevant secondary task. As a result, based on this finding, we would not expect transfer to happen. The present study trained subjects with an orientation discrimination task and a novel object categorization task in alternating game levels, each in a different retinal location pair in a dynamic, challenging environment. Nevertheless, it is possible that the two tasks interact so that the presence of a second task, with different stimuli at different retinal locations, would influence the amount of transfer to untrained conditions.

Our perceptual learning task was interleaved with other task irrelevant challenges (i.e. for example collecting coins and navigating), hereafter referred to as ‘game trials’. This could be seen as an analogue to what was done by Harris et al. [55] who aimed to eliminate adaptation effects by interleaving the perceptual task with task irrelevant trials. As a consequence, they obtained transfer of learning. By interleaving the perceptual learning task with irrelevant ‘game trials’, our paradigm might also reduce adaptation and thereby enhance transfer of learning.

Furthermore, a model proposed by Talluri et al. [56] illustrated the influence of the amount of training near versus supra threshold on the specificity of learning. Tedious training near threshold, or difficult tasks, led to asymptotic learning and specificity [3,4,29], whereas supra threshold training allowed for transfer. In the present study, as a result of a challenging game environment, the subjects were trained supra threshold. This can be derived from a comparison of the thresholds during training and gaming in Fig 4 and the thresholds during testing (without gaming) in Fig 5. Also this aspect of our design might foster transfer.

Literature on the specificity of learning is quite inconsistent, which in turn was attributed to a lack of power and the absence of an active control group [57]. The present study included an active control group, which also showed at least some learning. The presence of learning in both groups illustrates the importance of including an active control group to put the observed results in the experimental group in perspective. With regard to the object categorization performances, both groups improved across all conditions, however, learning was even more present in the perceptual learning game group.

Finally, the experimental paradigm included a feature-based expectation manipulation with regard to the stimulus orientation or object category of the upcoming stimulus. Throughout both perceptual learning tasks, valid expectations failed to improve performances compared to invalid expectations. According to the classic ideal observer framework, feature-based expectation could not lead to increased decision sensitivity, but gave rise to increased response bias. However, skepticism of this framework arose, with some claiming that feature-based expectations do alter detection sensitivity and are not the result of a response bias. However, among skeptics, there is no consensus either. One group of researchers argued that expectation altered the sensory signal [58], whereas a second group claimed that it changed the decision criterion [59–62]. Within the scope of this study, we did not untangle these two latest hypotheses, but we were able to show improvements from pre–to post training in the behavioral near transfer task set, which did not include an expectation manipulation. So the improvements in performance throughout training were not driven by a response bias, and also reflect an improved detection sensitivity.

### Far transfer

The present study assessed transfer of learning after training to different domains: bottom-up attention, namely selective attention in time, space [63] and towards objects [44], sustained attention [46], mental rotation [48], multitasking [49], and cognitive failures [50]. For three out of six tests, there was a general effect of learning in both groups in our study. Irrespective of the training regimen, all subjects improved their executive functioning, mental rotation performance, and multitask performance and speed. With regard to the non-significant findings, subjects remained at the same level when tracking multiple objects, sustaining attention, had similar amounts of impulsivity and reported the same amount of daily cognitive malfunctions. These findings were not what we would expect from the meta-analysis of Bediou et al. [25]. They stated that “action video game play robustly enhances the domains of top-down attention and spatial cognition, with encouraging signs for perception”. Given that none of the improvements were specific to the experimental group, and were equally large in the active control group, these findings are in line with Sala and Gobet [33].

Already in the year 1901, researchers claimed that near transfer has a fairly high chance of success, but that the opposite was true for far transfer [64]. More recently, a couple of meta-analyses shed a light on studies from the sixties up till now looking at improvements after video game interventions or in existing player groups [25,34,65–67]. These meta-analyses each focused on separate -some similar- moderating effects of video game play on improvements in the cognitive domain. Three important issues were shared: the importance of an active control group, the publication bias, and the video game genres. First, an active control group was argued to be a golden standard in all future training studies by Klingberg [68]. Yet, not many intervention studies included this golden standard. Some studies made use of a passive-control group, but compared to subjects in the experimental group, they were not involved in some kind of training. However, trained subjects knew that they received training, which was coupled with the belief that they should improve, which in turn could already impacted the results. The present study illustrated the importance of an active control group. Both groups benefitted from their training paradigm. Given that both groups participated in the same tests before and after training, the test re-test effect is expected to not influence the differential learning effects. The same is true for possible expectation effects, which showed to not line up between an experimental group and active control [69], and thus likely do not bias the results. There were no significant learning differences in far transfer between both groups, but few improvements were observed with training. A passive control group would allow for a more fine-grained investigation of these results. Consequently, this brings us to the second raised issue, the publication bias.

The Achilles heel of video game research is to be found in studies comparing an existing video game player group with a group of game novices. These studies were more prone to bias than intervention studies. More strikingly, the higher the journal’s impact, the larger the effect sizes and larger effect sizes in research groups focused on this kind of research compared to others [65]. The danger was not gone with the use of intervention studies, as suggested by Bediou [25] and Sala[33]. However, the present study aimed to report all findings, irrespective of the significance, which would help to alleviate the inconsistent results by providing the full picture.

A third raised issue, taken responsible for inconsistent findings, was the video game genre [70,71]. As a general principle, the majority of research studies included action video game playing. These kind of games are characterized by increased processing speed, quick and unpredictable events, a high cognitive load, and peripheral processing [72]. The biggest effect of training was found in action video game play, however, other studies showed a link between improvements in cognitive abilities and game genre suggesting that the specificity of video game play might be related to the performed actions during the game [71]. Consequently, action video game play might lead to enhancements in domains close to the trained domains with game training. On the other hand, Toril [66] reported no significant differences between simple and complex games with regard to cognitive improvements. Moreover, simple over complex games showed a non-significant trend towards more profitable gains. Our experimental game could be categorized as a simple game with more demanding challenges than the control game. This difficulty gap did not result in significant differences between the groups.

### Limitations and further directions

The majority of previous intervention studies paired with active control group were subjected to thorough meta-analyses. In this section, the focus is on the observed effect sizes, power and duration of training. First, some meta-analyses reported promising effect sizes (e.g., domains of spatial cognition, top-down attention and perception) [25], while others found relatively small ones (i.e. overall cognition) [33], which after publication bias analyses disappeared. There was some critique regarding the (zero) degree of heterogeneity between the effect sizes, such that variability between studies was likely the result of a sampling error. The discrepancy between positive [25] and near zero effect sizes might be due to more strict inclusion criteria and better analysis techniques used by Sala and Gobet. Second, meta-analytic power analyses pointed to underpowered studies [25], such that most studies only included 13–45 participants. They calculated that a correlation of .5 between pre- and post-tests, with only 15 subjects in each group, would need an effect size of .45 to obtain 80% power. Consequently, if the acquired effect size would only be a third of .45, 29 subjects are needed in each group. Within the present study, two groups with each 16 subjects were trained and yet both groups showed some degree of near and far transfer of learning, although not in each assessed domain. We could argue that the most prominent effects were found and that an increased sample size could reveal the presence of minor effects too. Third, the training duration meta-analytic findings, are not in line with each other. On the one hand, there is advocacy for more training which leads to increased effect sizes [25]. While, on the other hand, Sala and Gobet [33] do not find hours of training to have a moderating effect. Furthermore, they argue that a positive relationship between training duration and effect size is needed for training to change cognitive performances. To conclude, the more hours of training, the bigger the expected effect size. A training duration of 10–20 hours already altered top-down attention, however more training might be recommended when evaluating changes in perception (30–50 hours) according to Bediou and colleagues [25]. Subjects in the current study were trained for ten hours, and already showed positive ‘near transfer’ in novel object categorization. However, we might find stronger effects, and more specific effects, with larger amounts of training. Alternatively, other domains such as top-down attention (i.e., multiple object tracking ability) did not change after ten hours of training with our paradigm, and also here it is possible that stronger effects could be found after longer training.

Future studies could disentangle all the learning building blocks, to reveal whether some are a necessity for obtaining transfer, and to find how they interact with each other. The present study included a multitude of factors, such as the interaction between two different tasks, interleaving these tasks with ‘game trials’, supra threshold training, and a feature-based expectation manipulation. However, it remains unclear to what extend these individual elements lead to transfer. Consequently, future studies could disentangle these factors and investigate their contribution to obtaining transfer and how they interact. Furthermore, it would be very interesting to evaluate near and far transfer changes at the level of the brain with this integrated paradigm.

### General conclusion

The novel dynamic perceptual learning paradigm had the potential to train novices on low and high level features, and to generalize to untrained high level features and/or retinal locations. The near transfer results were in line with recent findings and added evidence to the existing debate. There were no training specific improvements in the far transfer tasks, which was in line with the current meta-analysis.
